# New Potentiometric Screen-Printed Platforms Modified with Reduced Graphene Oxide and Based on Man-Made Imprinted Receptors for Caffeine Assessment

**DOI:** 10.3390/polym14101942

**Published:** 2022-05-10

**Authors:** Hisham S. M. Abd-Rabboh, Abdel El-Galil E. Amr, Abdulrahman A. Almehizia, Ahmed M. Naglah, Ayman H. Kamel

**Affiliations:** 1 Chemistry Department, Faculty of Science, King Khalid University, P.O. Box 9004, Abha 61413, Saudi Arabia; hasalah@hotmail.com; 2 Department of Chemistry, Faculty of Science, Ain Shams University, Cairo 11566, Egypt; 3 Pharmaceutical Chemistry Department, College of Pharmacy, Drug Exploration and Development Chair (DEDC), King Saud University, Riyadh 11451, Saudi Arabia; mehizia@ksu.edu.sa (A.A.A.); anaglah@ksu.edu.sa (A.M.N.); 4 Applied Organic Chemistry Department, National Research Center, Dokki, Giza 12622, Egypt; 5 Chemistry Department, College of Science, Sakhir 32038, Bahrain

**Keywords:** solid-contact ISEs, caffeine, molecularly imprinted polymers (MIPs), screen printed, potentiometry

## Abstract

Caffeine is a psychoactive drug that is administered as a class II psychotropic substance. It is also considered a component of analgesics and cold medicines. Excessive intake of caffeine may lead to severe health damage or drug addiction problems. The assessment of normal caffeine consumption from abusive use is not conclusive, and the cut-off value for biological samples has not been established. Herein, new cost-effective and robust all-solid-state platforms based on potentiometric transduction were fabricated and successfully utilized for caffeine assessment. The platforms were modified with reduced graphene oxide (rGO). Tailored caffeine-imprinted polymeric beads (MIPs) based on methacrylic acid (MAA) and ethylene glycol dimethacrylate (EGDMA) were prepared, characterized, and used as recognition receptors in the presented potentiometric sensing devices. In 50 mM MES buffer, the sensors exhibited a slope response of 51.2 ± 0.9 mV/decade (*n* = 6, *R^2^* = 0.997) over the linear range of 4.5 × 10^−6^–1.0 × 10^−3^ M with a detection limit of 3.0 × 10^−6^ M. They exhibited fast detection of caffeinium ions with less than 5 s response time (<5 s). The behavior of the presented sensors towards caffeinium ions over many common organic and inorganic cations was evaluated using the modified separate solution method (MSSM). Inter-day and intra-day precision for the presented analytical device was also evaluated. Successful applications of the presented caffeine sensors for caffeine determination in commercial tea and coffee and different pharmaceutical formulations were carried out. The data obtained were compared with those obtained by the standard liquid chromatographic approach. The presented analytical device can be considered an attractive tool for caffeine determination because of its affordability and vast availability, particularly when combined with potentiometric detection.

## 1. Introduction

Caffeine (1,3,7-trimethyl xanthine) is an alkaloid, commonly used as an ingredient in many foods, beverages, and medicines. It is widely consumed as a psychoactive substance and is commonly found in coffee, tea, chocolate, cocoa, and soft drinks. Depending on the dosage, caffeine can have positive or harmful effects on the consumer [1,2]. Caffeine is a psychoactive drug that is administered as a class II psychotropic substance. It is also considered a component of analgesics and cold medicines. It is widely consumed as an anesthetic as it acts to stimulate the central nervous system [3], stimulate gastric acid secretion [4], diuresis [5], and increase blood pressure [6]. Excessive caffeine consumption has serious effects on human health such as dehydration [7], prevention of DNA repair [8,9], prevention of mineral absorption, especially iron [10], cancer [11], heart disease [12], aging, and pregnancy problems [13]. The concentration of caffeine varies depending on the type and nature of the products consumed. In coffee, its concentration ranges from 36 to 804 mg/L; in chocolate, it ranges from 17 to 551 mg/L; in iced tea drinks, it ranges from 13 to 68 mg/L; in sports drinks, it ranges from 267 to 340 mg/L; and in coffee-based drinks, it ranges from 15 to 448 mg/L; and in dietary supplements, it ranges from 1002 to 1353 mg/L [14,15]. In the USA, the average caffeine consumption among adults between 1994 and 2005 rose from 196 mg/day to 211 mg/day, while the global average consumption is around 70 mg/day [16,17,18]. Drug abusers often mix caffeine with sodium benzoate to snort it, or they take it with other illicit drugs to enhance the stimulating effect. From here, the need to monitor potential caffeine abuse among those with a history of drug addiction and abuse is an important task. The excessive intake of caffeine may lead to severe health damage or drug addiction problems [19,20]. However, unlike traditional illicit drugs, the assessment of normal caffeine consumption from abusive use is not conclusive, and the cut-off value for biological samples has not been established [21,22].

Of all the above, it is imperative to control the concentration of caffeine and determine its quantity. The challenge of quantifying caffeine is associated with its low concentration in complex matrices. As a result, most current caffeine quantification techniques have their limitations and disadvantages. There are many techniques such as spectrophotometry [23], fluorometry [24], capillary electrophoresis [25], gas chromatography [26], high-performance liquid chromatography (HPLC) with UV or diode detection methods [27] and mass spectrometry [28,29,30,31,32,33,34,35]. Disadvantages of these techniques include the high cost of the apparatus used, complexity in technologies, more time-consuming, low sensitivity, and selectivity.

Electroanalytical methods of analysis have good characteristics, including low instrument cost, simplicity of operation, high sensitivity and selectivity, wide dynamic linear range, real-time determination, miniaturization, convenience, and ease of use [36,37]. However, as a serious limitation in voltammetric techniques, caffeine can be oxidized at high positive potentials. This may interfere with the electro-oxidations of other species that coexist in the background electrolyte [38,39,40,41].

Ion-selective electrodes (ISEs) are considered indispensable members of the family of electrochemical sensors with the ability to measure basic ionic species in complex sample matrices [42,43,44]. Potentiometry with ISEs is characterized as a non-destructive and passive analysis method that converts the ionic activity into potential without the need for additional stimulation. In addition, they are characterized by their simplicity, low cost, ease of miniaturization, timeliness, and reliability. ISEs have been widely adopted as an accurate option for rapid ion detection in multiple fields such as biomedicine [44,45,46], agriculture [47], environmental monitoring [48,49], and industrial analysis [43,50]. In recent years, rapid identification of ions ranging from environmental monitoring to bio-fluid analysis at the point of care has required powerful and robust analytical tools. This was driven by the development of materials science and processing technology. Solid-contact ion-selective electrodes (SC-ISEs) have shown great potential for routine and portable ion detection [51,52]. Introducing nanomaterials as ion-to-electron transducers with the adoption of various strategies to improve performance led to a significant boost in the development of SC-ISEs [53]. Besides, with the increase in miniaturization, flexibility, and reliability of SC-ISEs, the field has developed from the traditional potentiometric electrodes to the integrated sensing systems which have wider applications. Now, screen printing technology has been widely used to manufacture a new generation of electrochemical sensors characterized by their low cost, ease of use, and ease of disposal. This technology contributed greatly to establishing the route from the ‘laboratory to the market’. Due to their beneficial physical properties, such as disposability, simplicity, and rapid responses, these types of platforms have been successfully used for rapid analysis of both environmental pollutants [54,55] and biomedical molecules [37,56].

Molecularly imprinted polymers (MIPs) have seen a continuous development as sensing elements in bio-/chemo-sensors since the late 1990s [57]. MIPs are attractive not only for their recognition properties that are close to those of natural receptors and their availability for a wide range of targets but also for their superior chemical and physical stability compared to biological receptors. The field of molecularly imprinted polymer (MIP)-based chemosensors has been experiencing constant growth for several decades [58,59,60]. Since the beginning, their continuous development has been driven by the need for simple devices with optimum selectivity for the detection of various compounds in fields such as medical diagnosis, environmental and industrial monitoring, food, and toxicological analysis, and, more recently, the detection of traces of explosives or their precursors [61,62].

In this work, cost-effective, compact, and portable monitoring potentiometric sensors were manufactured. These sensors have been successfully applied to determine caffeine in different matrices. All-solid-state screen-printed potentiometric electrodes were fully designed, characterized, and proposed. The potentiometric analytical device integrates the indicator polymeric membrane caffeine-ISE with an Ag/AgCl reference electrode and a polyvinyl butyral (PVB) reference membrane. All potentiometric performances of the fabricated screen-printed sensors were investigated and evaluated.

## 2. Materials and Methods

### 2.1. Apparatus

All potential measurements were carried out using a mV/pH meter (PXSJ-216 INESA, Scientific Instrument Co., Ltd., Shanghai, China). Carbon screen-printed electrodes (SPEs) modified with multiwalled carbon nanotubes (MWCNTs) (Ref. 110CNT) were purchased from DropSens (Llanera, Asturias, Spain). The platforms were of ceramic substrate (L34 × W10 × H0.5 mm) and silver electrical contact. Millipore Milli-Q system (18.2 MΩ cm specific resistance) was used for obtaining de-ionized water. A scanning electron microscope (SEM) (JEOL JSM 6510lV, Osaka, Japan) was used for the characterization of MIP particles.

### 2.2. Reagents and Chemicals

High molecular weight poly (vinyl chloride) (PVC), 2-nitrophenyl octyl ether (o-NPOE), polyvinyl butyral (PVB), 2-(N-morpholino) ethane sulfonic acid (MES), caffeine, potassium tetrakis (4-chlorophenyl) borate (KTClPB), methyl acrylic acid (MAA), benzoyl peroxide (BPO) and ethylene glycol di-methacrylate acid (EGDMA 98%) were purchased from Sigma Aldrich (St. Louis, Missouri, MO, USA). Tetrahydrofuran (THF) and acetonitrile were obtained from Fluka AG (Buchs, Switzerland). Ag/AgCl ink (E2414) was purchased from Ercon (Wareham, MA, USA). Tablets containing caffeine were purchased from different Egyptian pharmacies. They were represented as Spasmomigran (Kahira Co., Cairo, Egypt), Cafergot (Novartis Co., Cairo, Egypt), Amigraine (Adco Co., Cairo, Egypt), and Samadril (Minaph Co., Egypt).

A stock 1.0 × 10^−2^ M caffeine solution was prepared by dissolving the definite weight in 100 mL de-ionized water. All caffeine standard solutions (e.g., 1.0 × 10^−7^–1.0 × 10^−2^ M) were prepared from the stock caffeine solution after dilution with 50 mM MES buffer solution of pH 5.0. All solutions were stored in the refrigerator.

### 2.3. Preparation of the Imprinted Beads

The imprinted polymers were prepared by mixing 0.5 mmol of caffeine (template) with 3.0 mmol of MAA (functional monomer) in a glass tube and left together for 1 h. 3.0 mmol of EGDMA (cross-linker) was then added and followed by adding 0.3 mmol of BPO (initiator). 15 mL of acetonitrile (porogenic solvent) was added to the mixture followed by sonication for 10 min until complete dissolution of all components. The solution was degassed with N_2_ gas for 15 min to expel all dissolved oxygen. The solution mixture was placed in an oil bath for 18 h at 70 °C for complete polymerization. The synthesized polymers were washed with methanolic solution till all un-reacted reactants were removed. Using Soxhlet, the templated caffeine molecule was removed from the MIP particles using a mixture of methanol/acetic acid (8:2, *v*/*v*). The washing solution was measured spectrometrically at λ_max_ = 250 nm to check the complete removal of caffeine from the MIP particles. The particles were washed several times until there was no further detection of caffeine. The obtained MIPs were dried at ambient temperature before use. Non-imprinted polymers (NIPs) were also prepared in a similar way as the imprinted beads but with the exclusion of caffeine from the procedure.

### 2.4. Sensor Fabrication

**Figure 1:**
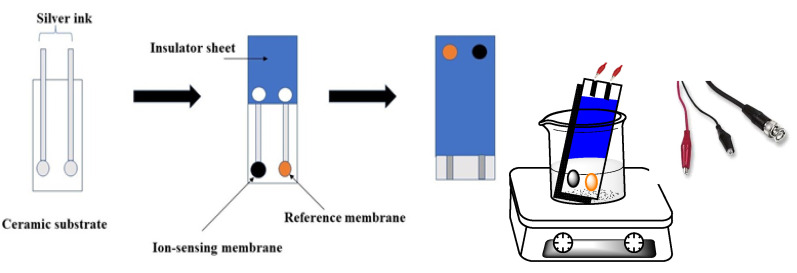
Schematic design of homemade screen-printed fabrication.

The composition of the ion-sensing membrane (ISM) was of a total mass of 114 mg dissolved in 2.0 mL THF. The membrane was prepared via dispersing MIP or NIP particles (12 mg), 2 mg of (rGO), KTpClB (2.0 mg), plasticizer (49.0 mg), and PVC (49.0 mg). For the modified screen-printed electrodes (SPEs), about 5 μL of the ISM cocktail was applied onto the carbon orifice of the SPEs via the drop-casting method and left to dry. The membrane composition of the reference electrode contains 70 mg of NaCl and 78.1 mg of polyvinyl butyral (PVB) dissolved in 1 mL of methanol. A 20 µL of the reference membrane solution was drop cast on the Ag/AgCl ink electrode surface. The solid-state Ag/AgCl reference electrode was integrated with the potentiometric sensor into the screen-printed platform. A schematic representation of the designed sensors is shown in Figure 1.

## 3. Results and Discussion

### 3.1. MIPs Characterization

**Figure 2:**
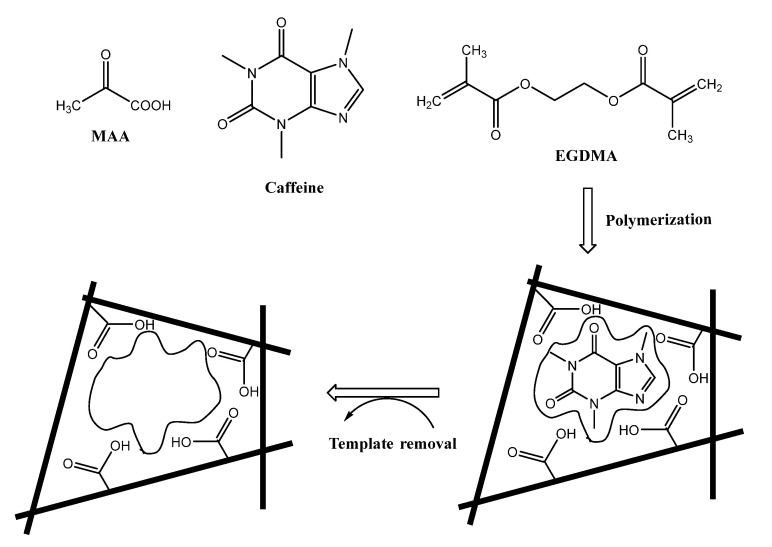
A schematic representation for the imprinting process.

The caffeine-imprinted beads were synthesized using methacrylic acid (MAA) as an afunctional monomer and ethylene glycol dimethacrylate (EGDMA) as a cross-linker in the ration 0.5: 3: 3 for the template, monomer, and cross-linker, respectively. In the imprinting process, the interaction between the functional monomer and the templated molecule can be accomplished through: (1) Hydrogen bond formation between the carboxyl group (COOH) in MAA with either the carbonyl group or the tertiary amine in caffeine; (2) A π–π interaction between the π- bonds in the caffeine molecule and carbonyl groups in both MAA and EGDMA. These types of interactions enhance the binding affinity and specificity of the MIP towards caffein recognition [63]. Figure 2 represents a representation pathway for the imprinting process.

**Figure 3:**
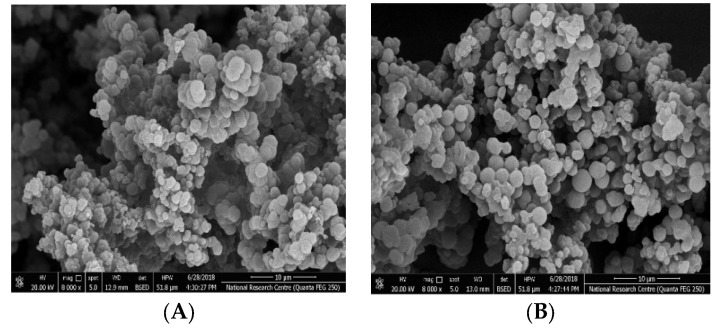
SEM images of (**A**) MIPs and (**B**) NIPs.

An investigation of the surface morphology of the synthesized polymeric beads was carried out using the scanning electron microscopy (SEM) technique. For the MIP beads, the SEM pictures showed a uniform, regular and semi-spherical shape with an average diameter of 300 nm. These beads were well dispersed in the plasticized PVC membrane. They could reduce the membrane resistance of the sensing membrane and create more recognition sites inside the membrane [63]. As the NIP beads were synthesized in a similar way as MIP beads but without a caffeine template, the morphological structure revealed a more regular, uniform, and spherical shape than MIPs beads. This can be attributed to the presence of caffeine template in the MIP beads. The SEM images for both MIPs and NIPs are presented in Figure 3.

### 3.2. Sensors’ Characteristics

**Figure 4:**
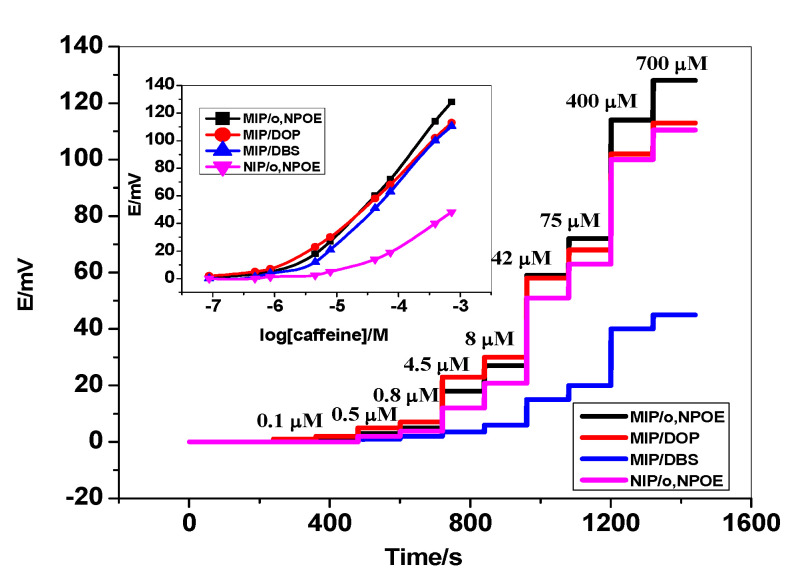
Calibration plot for caffeine sensors in different membrane plasticizers.

The all-solid-state sensors based on either MIPs or NIPs exhibited a potentiometric response towards caffeine within the concentration range of 1.0 × 10^−7^ to 1.0 × 10^−3^ M caffeine at pH 5 (50 mM MES buffer). The time trace versus the potential response with the corresponding calibration curves is shown in Figure 4. For membrane composition optimization, different plasticizers namely o, NPOE, DOP, and DBS were examined. For sensors based on MIPs plasticized in o,NPOE, they exhibited a Nernstian response with a slope of 51.2 ± 0.9 mV/decade (*n* = 5, *R^2^* = 0.997) over the concentration range of 4.5 × 10^−6^ to 1.0 × 10^−3^ M and a detection limit of 3.0 × 10^−6^ M. Sensors based on MIPs plasticized in DOP exhibited a Nernstian response with a slope of 43.6 ± 0.5 mV/decade (*n* = 5, *R^2^* = 0.999) over the concentration range of 7.7 × 10^−6^ to 1.0 × 10^−3^ M and a detection limit of 4.0 × 10^−6^ M. The sensors’ membrane plasticized in DBS exhibited a potentiometric response with a slope of 45.4 ± 1.3 mV/decade (*n* = 5, *R^2^* = 0.999) over the concentration range of 8.0 × 10^−6^ to 1.0 × 10^−3^ M and a detection limit of 4.5 × 10^−6^ M. For sensors based on NIPs, they exhibited a sub-Nernstian response with a slope of 28.1 ± 0.9 mV/decade (*n* = 5, *R^2^* = 0.997) over the concentration range of 4.5 × 10^−5^–1.0 × 10^−3^ M, and a detection limit of 1.7 × 10^−5^ M. This can confirm the existence of binding sites in MIPs’ particles in the ion-sensing membrane. All the analytical features and the potentiometric response of the proposed sensors are summarized in Table 1.

### 3.3. Transduction Mechanism

**Figure 5:**
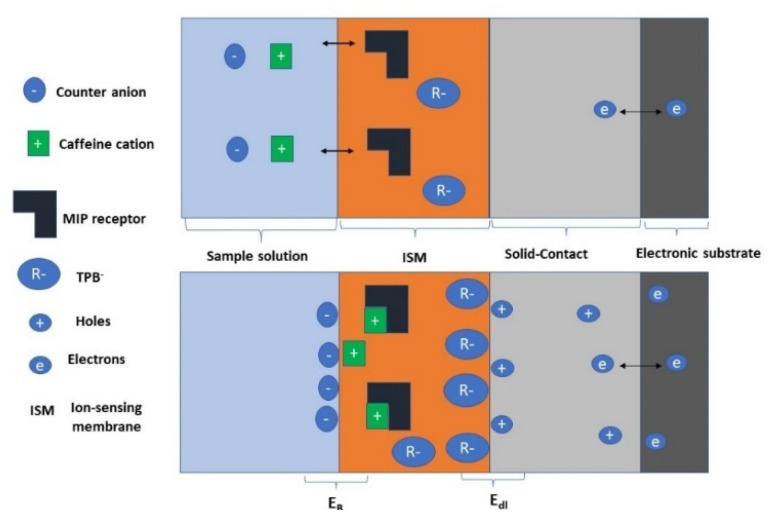
Response mechanisms for the SC-ISEs. Electric-double-layer (EDL) capacitance-based SC-ISEs with MWCNT and rGO as solid-contact transducer.

In this work, both MWCNTs and rGO were introduced together as solid-contact materials during the manufacturing of the presented sensors. They can be considered as high surface area nanostructured materials that exhibit their ion-to-electron transduction properties, forming the electrical double layer at the polymeric ISE membrane/solid contact interface. The overall reaction includes mainly three equilibrium charge transfers at three boundaries or interfaces. At first, the reaction at the E-conducting substrate and solid-contact interface exhibits the electron transfer reaction. Secondly, an electrical double-layer (*E_dl_*) is formed between cations or anions coming from the ISE membrane and the electrical charges (either electrons or holes) that were formed in the porous structure of the solid-contact material. The potential at the E-conducting substrate and solid contact interface is very small (*E ≈ 0*). So, smost E_DL_ capacitance-based SC materials are considered highly electronic conductive. At the solid contact/ion-selective membrane interface, there is no charge transfer reaction, but the exhibited potential was declined to double-layer capacitance as shown in Figure 5. Thirdly, an interfacial phase-boundary potential is formed at the interface between the attached ion-selective membrane and the aqueous solution. Thus, the exhibited potential *E_B_* confirms the Nernstian response toward the desired ion and emphasizes the thermodynamical potential of double-layer capacitance (*C_dl_*)-based solid-contact ISEs.

### 3.4. Method Validation

Reliability, quality, and consistency of results are important features and evidence of analytical method validation that ensure results fitting for the same method under the same set of conditions and control parameters [64]. There are a number of ways to classify the method validation as the following:

#### 3.4.1. Limit of Detection (LOD) and Linearity

The LOD parameter is defined as the lowest concentration noise with a certain degree of confidence. There are several ways to distinguish LOD depending on the signal-to-noise ratio. But in the case of potentiometric analysis, LOD can be determined from the cross point of the lines fitted to the linear segments of the *emf* vs. log *a_i_* curve as shown in Figure 4. The LOD is not a robust or rugged parameter and can be affected by minor changes in the analytical system (e.g., temperature, matrix effects, purity of reagents, instrumental conditions). Linearity definition exhibited from obtained signals, which are directly proportional to the concentration of analyte in the sample. The performance characteristics of the MIP/o,NPOE sensors exhibited a wide and linear dynamic range between 1.0×10^−3^–4.5 × 10^−6^ M with near-Nernstian slopes of 51.2 ± 0.9 mV/decade. The calibration plot with regression equation was found to be Y (mV) = 51.2 log [caffeine] + 131.6 with a correlation coefficient of 0.999 between the standard caffeine concentration and the potential measured in triplicates (*n* = 3). The detection limit of the presented sensor (DL) was found to be 3.0 × 10^−6^ M.

#### 3.4.2. Reproducibility and Repeatability

The reproducibility (between-run or inter-assay variation) and repeatability (within-run or intra-assay variation) were evaluated for the proposed sensor. A standard caffeine sample (10 µg/mL) was measured to carry out these tests. The intra- and inter-day precision was below 0.59 and 0.84%, respectively. This confirmed the agreement between the results obtained by measuring the caffeine reference sample under different conditions with different sensor assemblies and different mV/meters at different times.

#### 3.4.3. Trueness, Bias and Recovery

The meaning of trueness relates to the systematic error of a measurement system that is considered a closeness of agreement between the average of an infinite number of replicates measured quantity values and a reference quantity value. Bias definition is the agreement between the mean value of replicate measurements and the true value of the measured quantity [64].

Trueness and bias of the proposed sensor were examined by using six replicate measurements of 10 µg/mL caffeine as an internal quality control sample. Their values were calculated as in Equations (1) and (2):

Trueness% = X/µ × 100(1)

Bias% = (X − µ)/µ × 100(2)

where X is the mean of test results obtained for the reference sample and μ is the true value given for the reference sample.

The obtained trueness and bias were found to be 99.3 and 0.7%, respectively.

The recovery (%) of spiked caffeine samples was tested using six replicate measurements of spiked 1.5 µg/mL caffeine. The recovery (%) was found to be 99.4%.

#### 3.4.4. Ruggedness and Robustness

**Figure 6:**
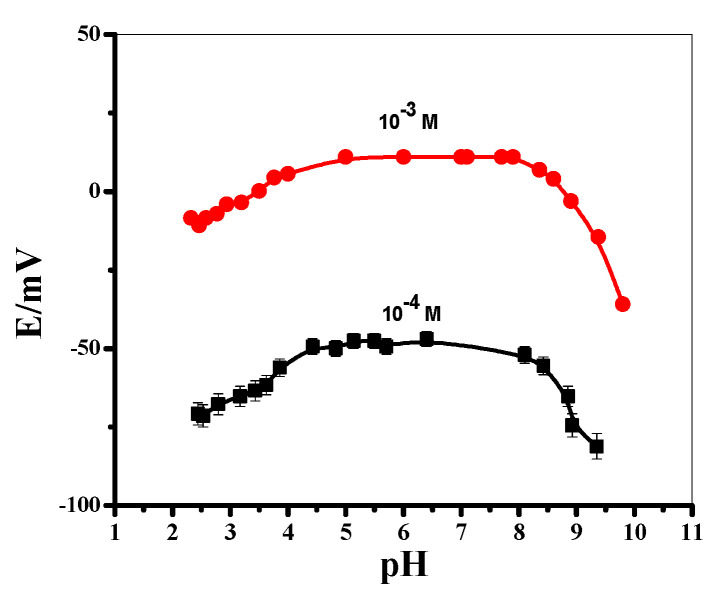
The effect of pH on the potentiometric response of the MIP/oNPOE membrane-based sensor.

The term ruggedness in analytical methodology is considered the degree of reproducibility of obtained results of the same sample under varying test conditions. On the other hand, robustness is defined as the stability of the method against small variations of the intrinsic method parameters and variability of the sample matrix [64]. The potentiometric features of the proposed sensor were examined over a wide range of variable pH values [e.g., 2–10]. The test was performed using two concentrations of caffeine (10^−3^ and 10^−4^ M). The pH of the test solutions was adjusted after adding small aliquots of HCl and/or NaOH. The obtained potentiometric response of the presented sensor was recorded with its corresponding pH value of the test solution. Since the *pKa* of caffeine is 14.0, therefore caffeine will be in its cationic form at pH below this *pKa* value. As shown in Figure 6, it exhibited a different pH influence on the applied sensor properties that revealed the range of its stability over the pH range 4.3–8.5. At pH < 5.3, the potential response quickly declined. This can be attributed to the existence of di-valent or tri-valent caffeine cations in the solution that can decrease the potentiometric response. At pH > 8.5, the potential response quickly declined which can be attributed to the decrease of caffeine cation concentration due to the formation of some non-ionized caffeine. This wide range of stable potential readings revealed that a sophisticated, rugged, and durable potentiometric sensor was investigated. For further potentiometric studies of the applied sensor, a MES buffer of a 50 mM (pH 5) was chosen to be a working pH throughout.

### 3.5. Sensors’ Selectivity

The various interference attitudes that can present in the matrices during caffeine determination was investigated. The selectivity of the caffeine sensor was evaluated using the method presented by Bakker [i.e., the modified separate solution method (MSSM)] [65]. Different interfering species were tested which can coexist with caffeine in either its pharmaceutical forms or biological fluids. Among these species, there are salts of Na^+^, K^+^, Mg^2+^, and Ca^2+^ as cationic species. In addition, amino acids (alanine, arginine, and glycine), sugars (glucose and lactose), nicotine, chlorpheniramine, ephedrine, codeine, paracetamol, creatinine, camylofine and aspirin were added. The selectivity coefficients (*log K ^pot^_caffeine, J_*) were calculated and are shown in Table 2. It was shown that there is no significant interference from the interfering ions on the potentiometric response of the presented sensor. The sensors offered high selectivity and high efficiency towards the determination of caffeine in real samples.

### 3.6. Chronopotentiometry and Electrochemical Impedance Spectroscopy (EIS) Measurements

**Figure 7:**
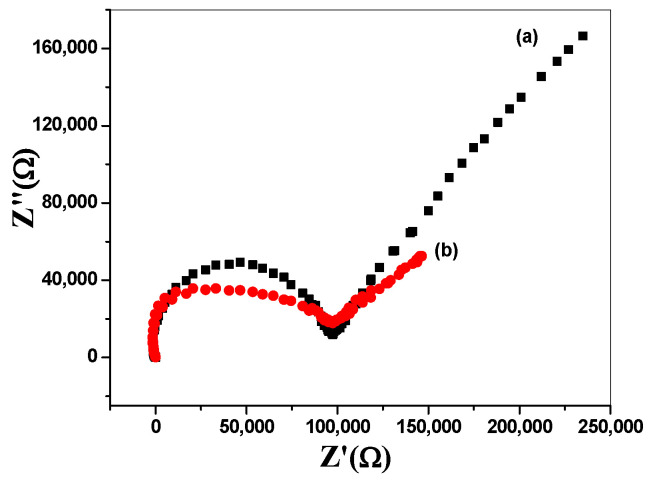
EIS measurements of (**a**) non-modified and (**b**) modified caffeine-ISEs.

To evaluate the double-layer capacitance and membrane resistance, EIS measurements were carried out. The measurements were performed by using a one-compartment three-electrode cell using (NOVA 2.0 software; Metrohm Auto lap B.V. Utrecht, The Netherlands) attached with a reference electrode (Ag/AgCl/KCl (3 M) and Pt auxiliary electrode. The applied frequency range starts from 100 kHz to 0.1 Hz using a sinusoidal excitation signal with excitation amplitude of 10 mV. The applied method was carried out by using a solution of 10^−3^ M of caffeine in a 50 mM MES buffer, pH 5. As shown in Figure 7, the method exhibited the Nyquist plots (complex plane plots of –Z^\\^ vs. Z^\^) on the equivalent circuit models. The bulk resistance (*R_b_*) of both the modified and non-modified electrodes were 0.1 ± 0.03 and 0.09 ± 0.0002 MΩ, respectively. The double-layer capacitances (*C_dl_*) were measured at the low-frequency branch (semicircle) for both the modified and non-modified sensors and were found to be 9.3 ± 1.1 and 22.5 ± 1.4 µF, respectively.

**Figure 8:**
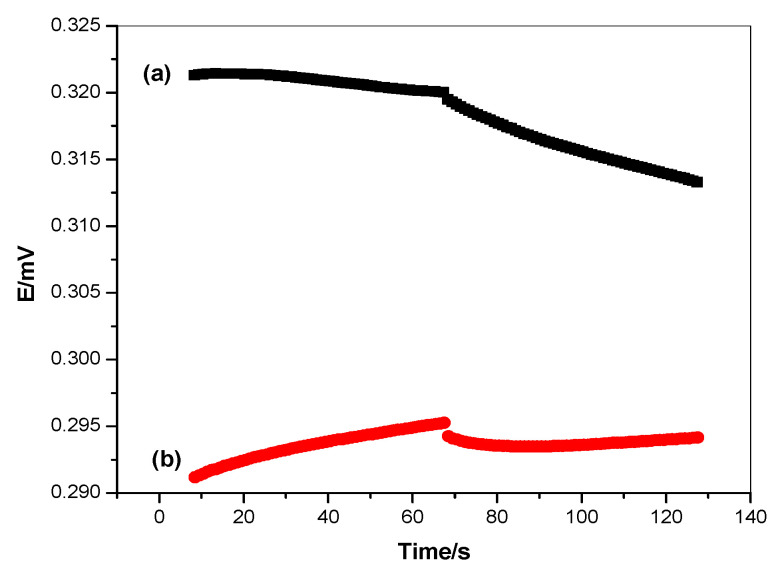
Chronopotentiometry measurements of the applied sensor; (**a**) non-modified and (**b**) modified caffeine-ISEs.

Short-term potential stability was evaluated via reverse-current chronopotentiometric measurements suggested by Bobacka [66]. The applied current was ± 1nA in both the anodic and cathodic directions for 60 s. The chronopotentiograms for both modified and non-modified sensors are shown in Figure 8. The potential drift *(∆E/∆t)* was found to be 97.7 and 41.6 µV/s for both the non-modified and modified caffeine sensors, respectively. These data show the incredibly increasing potential stability in the presence of the transducers. The capacitance double-layer [*C_L_ = I/(∆E/∆t)*] was 10.2 ± 0.5 and 24.2 ± 0.7 µF for both the non-modified and modified caffeine sensors, respectively. All data obtained via the EIS and chronopotentimetric measurements reflect the effect of the lipophilicity of the solid-contact transducing material and the highest double-layer capacitance formed upon the insertion of the rGO layer between the ion-sensing membrane and the electronic conductor substrate. The results confirmed that the rGO based sensor type revealed high potential stability, good conductivity, and high compatibility with the caffeine membrane-based sensor for the determination of caffeine in its matrices.

### 3.7. Water-Layer Test

**Figure 9:**
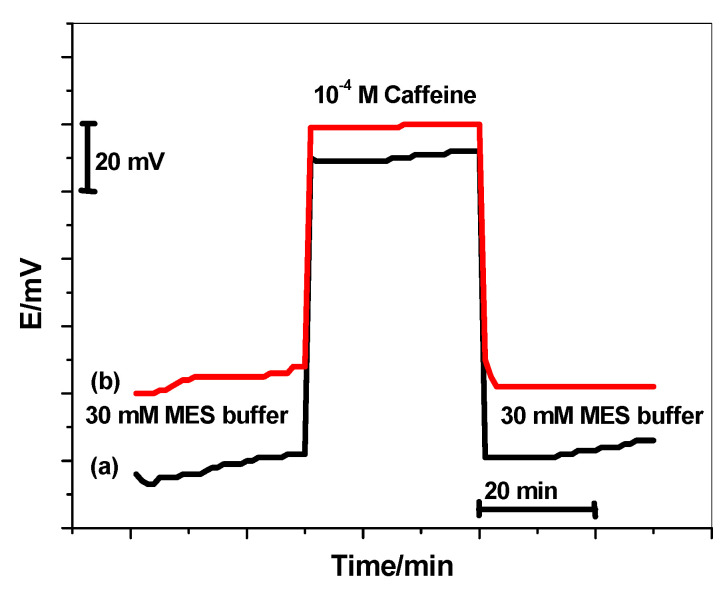
Water-layer tests for (**a**) non-modified, and (**b**) modified caffeine-ISEs.

The prejudicial effect to the potential stability and lifetime of the proposed sensor was also investigated. The test was applied with both the sensors present and absent in the solid-contact materials. The sensors were first immersed in 30 mM of the MES buffer (pH 5) for 30 min and the potential was recorded during this interval of time. After that, they were immersed in 10^−4^ M of caffeine for another 30 min. and finally, they were immersed again in 30 mM of MES buffer (pH 5) for a further 30 min. As shown in Figure 9, there is an enhanced potential-stability for caffeine sensors when they were modified with the lipophilic rGO layer. There is a potential drift for the non-modified sensors. This reflects the existence of water-layer formation between the ion-sensing membrane and the electronic-conducting substrate.

### 3.8. Caffeine Assay in Different Pharmaceutical Formulations

Caffeine is present in different pharmaceutical dosages like tablets and capsules. For caffeine stock preparation, 10 tablet contents were weighed and the mean weight of the active ingredient was calculated per one tablet. The accurate weight of the powder corresponding to 0.194 g of caffeine was dissolved in 100 mL 50 mM MES buffer, pH 5 to obtain 10^−2^ M caffeine stock. The solution mixture was sonicated for 1 h to ensure complete dissolution of the active ingredient. The solution was filtered and was diluted to several concentrations of caffeine (10^−3^–10^−5^ M). After constructing the calibration plot, the potential recorded for these samples was introduced to the regression equation of the calibration curve and the amount of caffeine was then calculated.

As shown in Table 3, the recoveries of caffeine measurements by using the proposed potentiometric method were of the range 95.2 to 106.5%. The results obtained were compared with those obtained by the HPLC standard method [67]. The recoveries of this method were of the range 99.0 to 100.8%. F and *t*-student tests were utilised for the two methods and revealed no significant difference between them, confirming the successful applicability of the proposed sensor for the determination of caffeine.

## 4. Conclusions

A new potentiometric method of caffeine determination in its solutions is presented through screen-printed carbon electrodes, modified with reduced graphene oxide (rGO) which were used for the fabrication of the sensors. Tailored caffeine-imprinted polymers (MIPs) were synthesized using methacrylic acid (MAA) monomer and this has been used as an electroactive receptor for caffeine. The sensors revealed a Nernstian response with a slope of 51.2 ± 0.9 mV/decade (*n* = 6, *R^2^* = 0.997) over the linear range of 4.5 × 10^−6^–1.0 × 10^−3^ M with a detection limit of 3.0 × 10^−6^ M. They exhibited fast detection of caffeinium ions with a response less than 5 s response time (<5 s). EIS and chronopotentiometric measurements were used to evaluate the potential-stability and double-layer capacity of the presented modified sensor. The double layer and potential drift of rGO based sensor were 24.2 ± 0.7 µF and 41.6 µV/s, respectively. The application to caffeine assessment in different pharmaceutical formulations was also successfully carried out. This reflects that the presented analytical device can be considered as an attractive tool for caffeine determination due to its affordability and vast availability, particularly when combined with potentiometric detection.

## Figures and Tables

**Table 1:** The analytical features and the potentiometric response of the proposed sensors.

Parameters	MIP/o,NPOE	MIP/DOP	MIP/DBS	NIP/o,NPOE
Slope (mv/decade)	51.2 ± 0.9	43.6 ± 0.5	45.4 ± 1.3	28.1 ± 0.9
Detection limit, (M)	3.0 × 10^−6^	4.0 × 10^−6^	4.5 × 10^−6^	1.7 × 10^−5^
Correlation coefficient (r^2^)	0.997	0.999	0.999	0.997
Linear range, (mol/L)	4.5 × 10^−6^ to 1.0 × 10^−3^	7.7 × 10^−6^ to 1.0 × 10^−3^	8.0 × 10^−6^ to 1.0 × 10^−3^	4.5 × 10^−5^–1.0 × 10^−3^
Response time, (s)	<5	<5	<5	<5
pH range	4.3–8.5	4.3–8.5	4.3–8.5	4.3–8.5
Precision, (%)	1.7	1.6	0.6	1.2
Accuracy, (%)	99.0	99.9	99.4	99.2
Standard deviation, (mv)	±2.4	±2.9	±1.2	±2.6

**Table 2:** The selectivity coefficients (*log K ^pot^ _caffeine, J_*) of the proposed sensor.

Interfering Ion	*log K ^pot^ _caffeine, J_* ±SD *
K^+^	−4.9± 0.1
Na^+^	−5.8 ± 0.4
Mg^2+^	−5.3 ± 0.3
Ca^2+^	−5.2 ± 0.1
Arginine	−5.1 ± 0.2
Alanine	−4.3 ± 0.3
Glycine	−4.6 ± 0.4
Nicotine	−3.3 ± 0.2
Glucose	−5.4 ± 0.1
Lactose	−5.2 ± 0.4
Aspirin	−3.3 ± 0.2
Codeine	−3.1 ± 0.7
Paracetamol	−3.4 ± 0.5
Creatinine	−3.7 ± 0.1
Camylofine	−2.9 ± 0.3
Ephedrine	−2.4 ± 0.1
Chlorpheniramine	−3.05 ± 0.3

* ±SD (standard deviation) (*n* = 3).

**Table 3:** Caffeine assessment in different pharmaceutical formulations using the proposed caffeine-based sensor.

Pharmaceutical Product and Source	Nominal Content Taken, mg/Tablet	Found, mg/Tablet ^a^				*^b^ F*-Test	*^b^ t*-Student Test
		Proposed Method	Recovery% ±SD	Reference Method [67]	Recovery% ±SD		
Pirafene caffeine, Memphis Co., Egypt	20	21.3 ± 0.6	106.5 ± 0.4	20.1 ± 0.4	100.5 ± 0.5	2.2	1.6
Allergex caffeine, Eipico Co., Egypt	50	48.9 ± 0.9	97.8 ± 0.6	49.5 ± 0.5	99.0 ± 0.7	9.3	0.3
Alertin, Pharco Co., Egypt	20	19.5 ± 2.2	97.5 ± 1.5	19.8 ± 0.2	99.0 ± 0.9	2.6	3.6
Vegaskine d, Alexandria Co., Egypt	25	23.8 ± 1.3	95.2 ± 1.1	25.2 ± 0.7	100.8 ± 0.2	6.7	2.2

*^a^* Mean of three replicate measurements ± standard deviation (SD), *^b^* F and *t-*Student tests at 95% confidence level values are 19.00 and 4.3, respectively.
